# Supplementary material for: HIV-positive status disclosure and associated factors among children in public health facilities in Dire Dawa, Eastern Ethiopia: A cross-sectional study
Source: PLoS One. 2020 Oct 12;15(10):e0239767. doi: 10.1371/journal.pone.0239767 (PMC7549787; doi:10.1371/journal.pone.0239767)
Supplement: S1 Questionnaire — (DOCX) [file pone.0239767.s001.docx]

**Amharic Information Sheet (Amharic Version)**

ይህ የሚካሄደው ልጆች ስለኤቻይቭ ኤድስ ሁነታ ስለማወቃቸዉ እና የተያያዙ ጉዳዮችን በድሬ ዳዋ ጤና ተቋማቶች ላይ የሚድረገዉ ጥናት ነው፡፡

የምርምር ፕሮጀክቱ ርዕስ፡ ልጆች ስለኤቻይቭ ኤድስ ሁነታቸዉ ስለማወቃቸዉ እና የተያያዙ ጉዳዮችን ለማወቅ የሚደረግ

**የዴርጅቱ ስም፡-** ድሬ ዳዋ ዩኒቨርሲቲ

የገንዘብ ዴጋፍ ያደረገው ዴርጅት ስም፡- ድሬ ዳዋ ዩኒቨርሲቲ

**መግቢያ**

የዚህ ምርምር ማብራሪያና የስምምነት ቅጽ አሊማ አሁን እርስዎ እንዱሳተፈበት የምንጠይቆትን የምርምር ጥናት ምንነት ማብራራት ነው፡፡ በዚህ የምርምር ፕሮጀክት ለመሳተፍ ከመወሰዎ በፊት ይህንን የማብራሪያ ቅጽ በጥንቃቄ በማንበብ ጥያቄ ካለዎት ይጠይቁ፡፡ በጥናቱ መሳተፍ ከጀመሩ በሁላ በማንኛው ጊዜ ጥያቄ ካለዎት መጠየቅ ይችላሉ፡፡

የምርምር ፕሮጀክቱ **ዓላማ**

የዚህ ጥናት ዓሊማ ልጆች ስለኤቻይቭ ሁነታቸዉን ያዉቃሉ በሚል እና ተያያ ጉዳዮዎችን ለመፈተሽ የሚደረግ ጥናት ነው፡፡

**የአሰራር ሂደት**

በዚህ ጥናት ውስጥ መሳተፍ ከተስማሙ ስምምነቱን በደንብ መረዲትና እንዱሁም መፈረም ይገባዎታሌ፡፡ ከዚህ በመቀጠል በጥናቱ መረጃ ሰብሳቢዎች ለሚጠየቁት ጥያቄ እንዱመልሱ ፍቃደኛነቶት ይጠይቃል፡፡

ሊከሰቱ የሚችሉ **ስጋቶቸና ምቾት መጓደልች**

በዚህ ጥናት መሳተፍዎ ምናልባት ጊዜዎትን ሉሻማዎት ይችል ይሆናል፤ ነገር ግን ወደ ጤና አገልግልት ሰጪ ድርጅቶች ከመመሊለስዎ እና የጥናቱ ውጤት ልጆች ስለበሽታቸዉ ሁነታ ስለማወቅ ከሚሰጠው ጥቅም አንፃር ይህን ያህል አይደለም፡፡ በዚህ ጥናት በመሳተፍዎ ምንም አይነት ስጋት (ችግር) አያጋጥምዎትም፡፡

**ጥቅሞች**

በዚህ ጥናት በመሳተፎ የተለየ ጥቅም አያገኙም ነገር ግን የእርሶ በጥናቱ መሳተፍ ልጆች ስለበሽታቸዉ ሁነታ ማወቅ እንዱሁም ያሉትን ችግሮችን ለማወቅ ይረዲል፡፡

**ማካካሻ**

በዚህ ጥናት በመሳተፍዎ ምንም አይነት ማካካሻ አይሰጦትም፡፡ ነገር ግን በጥናቱ በመሳተፍዎ ምስጋናችን ከፍተኛ ይሆናል፡፡

ሚስጢር ስለመጠበቀ

ከዚህ ጥናት የሚገኘው መረጃ ሁለ በሚስጥራዊነት ይጠበቃሌ፡፡ ለዚህ ጥናት የሚሰበሰበው እርስዎን የሚመለከት መረጃ በማህደር የሚቀመጥ ሲሆን ማህደሩም በስሞ ሳይሆን በተለየ ኮዴ ሲቀመጥ ኮደ ከዋናው ተመራማሪ ውጭ ለማንም አይገለጽም፡፡ በጥናቱ ያለመሳተፍ ወይም እራስዎን ለማግለል መብት: በጥናቱ ላለመሳተፍ ከፈልጉ በዚህ ጥናት ያለመሳተፍ እንዱሁም ከአንድ በላይ ወይም ሁሉንም ጥያቄዎች አለመመለስ ይቻላሉ፡፡ በዚህ ጥናት ባለመሳተፎ ወይም በከፊልም ሆነ በሙለ ጥያቄዎችን ባለመመለስዎ እንደነዋሪነቶ የሚያጡት አገልግልት አይኖርም፡፡

**የሚገናኟቸው ሰዎች**

ይህ ጥናት የጥናቱ ተሳታፉዎች ከጉዳት መጠበቃቸውን የሚያረጋግጠው ከድሬ ዳዋ ዩኒቨርሲቲ በሚገኘው ኮሚቴ ታይቶ ድጋፍ አግኝቶአል፡፡ በጥናቱ ዙሪያ ማንኛውም ጥያቄ ካልት የሚከተለትን ሰዎች በሚፈልጉት ጊዜ ማነጋገር ይችሊለ፡፡

1. አቶ አለሙ ጉታ፤ ድሬ ዳዋ፣ ስልክ ቁጥር: +251-945-99-64-59 ኢሜሌ ፣ chelsea0061@gmail.com

ይህንን ቅጽ አንብበው ከሆነ እና አሁንም ሆነ በላሊ ጊዜ ጥያቄ የመጠየቅ እዴል ተሰጥተው ከሆነ ወይም ይህ ቅጽ ተነቦና ተብራርቶልት ከሆነና ለመሳተፍ ከተስማሙ እባኮን ስሞንና ፉርማዎን ከዚህ በታች ያስቀምጡ፡፡

የተሳተፈበት ቀን ____________

የስምምነት ተቀባይ ፊርማ፡_____________________

የመጠየቁ ተራ ቁጥር ____________

| **ልጆች ስለኤቻይቭ ኤድስ ሁነታ ስለማወቃቸዉ እና የተያያዙ ጉዳዮችን በድሬ ዳዋ ጤና ተቋማቶች ላይ የሚድረገዉ ጥናት ነው፡፡ መጠየቅ በአማረኛ**  **ክፍል ሀ:** ሥነ-ህዝብና ማህበራዊ ጉዳዮች የተመለከተ መጠየቅ  **መመረያ**፡ መልሱ ላይ ይክበብ ወይም ይጠቀስ | | | | |
| --- | --- | --- | --- | --- |
| ተ.ቁ | ጥያቄ | ምላሽ | | |
| 001 | አድራሻ? | 1. ገጠር 2. ከተማ | | |
| 002 | እድሜዎ ምን ያህል ነው? | ………………በዓመት | | |
| 003 | ፆታ? | 1. ወንድ 2. ሴት | | |
| 004 | ሀይማኖት/ሽ ምንድነው? | 1. ኦርቶዶክስ 2. ሙስሊም 3. ፕሮትስታንት 4. ካቶሊክ 5. ለላ................................................ (መልሱ ይፃፊ) | | |
| 005 | ብሔርዎ መንድን ነዉ? | 1. አማራ 2. ኦሮሞ 3. ሶማሌ 4. ትግሬ 5. ጉራጌ 6. ለላ……………… | | |
| 006 | የትምህርት ሁነታ?  (መልሱ ላይ ክበበዉ) | 1. ማንበብ እና መፃፍ የማይችል 2. ማንበብ እና መፃፍ የሚችል 3. የመጀመሪያ ደረጃ 1-8 4. ሁለተኛ ና መስናዶ ደረጃ 9-12 5. ድፕሎማ እና ከዛበላይ | | |
| 007 | ስራዎት ምንድን ነው?  (መልሱ ላይ ክበበዉ) | 1. መንግስት ተቀጣሪ 2. የግል ተቀጣሪ 3. ተማሪ 4. ነጋዴ 5. የቤት እመቤት 6. ሌላ ካለ ይጠቀስ | | |
| 008 | የጋብቻ ሁኔታ ምንድን ነው?  (መልሱ ላይ ክበበዉ) | 1. ያላገባ 2. ያገባ 3. የተፋታ 4. የትዳር ጉደኛ የሞተበት | | |
| 009 | ከልጁ/ልጅቱዋ ጋር ያለሽ ግንኙነት ምንድን ነዉ? | 1. እናት/አባት 2. አያት 3. እህት/ወንድም 4. ዘመድ 5. ለላ ካለ ይጠቀስ | | |
| 010 | የልጁ/ልጅቱዋ እድሜዎ ምን ያህል ነው? | ………………በዓመት | | |
| 011 | የልጁ/ልጅቱዋ ፆታ? | 1. ወንድ 2. ሴት | | |
| 012 | የልጁ/ልጅቱዋ ትምህርት ሁነታ? | 1. ት/ቤት ያልገባ 2. የመጀመርያ ደረጃ 1-8 3. ሁለተኛ ደረጃና መስናዶ 9-12 | | |
| 013 | ልጁ/ልጅቱዋ በአሁኑ ሰዓት ከማን ጋር ነዉ የምኖረዉ/የምትኖረዉ? | 1. እናት/አባት 2. አያት 3. እህት/ወንድም 4. ዘመድ 5. ለላ ካለ ይጠቀስ | | |
| 014 | ልጁ/ልጅቱዋ ቤተሰቦቹዋን በኤድስ ሙቶባቸዋል/ሙተዋል? | 1. አዎ 2. አይደለም | | |
| 015 | ለጥያቄ 014 መልሱ አዎ ከሆነ ማነዉ? | 1. እናት ብቻ 2. አባት ብቻ 3. ሁለቱም 4. ወንድም/እህት | | |
| **ክፍል ለ፡**  ልጆች ስለ ኤችይቭ ኤድስ ስለማወቅ የተመለከተ ጥያቀ | | | | |
| ተ.ቁ | ጥያቄ | | መልስ | ይልፍ |
| 016 | ልጁ/ልጅቱዋ ኤችይቭ ኤድስ እንዳለበት/እንዳለባት ተነግረዋል/ተነግሮዋታል? | | 1. አዎ  2. አይደለም |  |
| 017 | ለጥያቄ 016 መልሱ አዎ ከሆነ በስንት ዓመቱ/ዋ ተነገረዉ? | | ………….ዓመት |  |
| 018 | ለጥያቄ 016 መልሱ አዎ ከሆነ ማን ነገረዉ? | | 1. እናት 2. አባት 3. አያት 4. እህት/ወንድም 5. ዘመድ 6. ጤና ባለሙያ 7. ጎደኛ 8. ለላ |  |
| 019 | ለጥያቄ 016 መልሱ አዎ ከሆነ ምክንያቱ ምንድን ነዉ? | | 1. ዕድሜዉ ስለደረሰ 2. በልጆች ተደጋጋሚ ጥያቄ 3. ልጁ/ልጅቱዋ ስለበሽታዎ አዉቀዎ መድሀኒቱን በአግባቡ እንዲወስድ/እንዲትወስድ 4. ስለ በሽታዉ ማወቅ መብቱ/ዋ ስለሆነ 5. ራሱን/ዋ እንዲከባከብ/እንዲትከባከብ እና ወደ ለላ ሰዉ እንዳይተላለፍ ማድረግ 6. ስለበሽታዉ አላፊነት እንድወስድ/እንድትወስድ 7. ለላ |  |
| 020 | ለጥያቄ 016 መልሱ አይደለም ከሆነ ያልተነገረዉ ምክንያቱ ምንድን ነዉ? | | 1. ዕድሜዉ ስላልደረሰ 2. ራሱን/ዋን እንዳይገልል ፈርቻ 3. ሚስጥር ስለማይጠብቅ 4. የወደፊት ሕይወቱ ከበተሰቦቹ ጋር ያለዉ/ያላት ግንኙነት እንዳይበላሽ ፊራቻ 5. ተስፋ እንዳያጣ/እንዳትጣ ፍራቻ 6. ቫይረሱን ስላሰተላለፍኩበት የጥፋትኝነት ስሜት ስለተሰማኝ 7. ከነገርኩት በሃላ የሚፈጠረዉ ድንጋጤ እና የጤና እክል ፍራቻ 8. ስለቫይርሱ እዉቀት ስለለኝ 9. ለላ…………………. |  |
| 021 | ለጥያቄ 016 መልሱ አይደለም ከሆነ (ካልነገርሽዉ) ጤና ተቆማት ለምን እዘሽ እንደምትመላለሽ ነገርሽዉ? | | 1. ለሳንቫ ነቀርሳ ህክምና ክትትል 2. ለልብ በሽታ ህክምና ክትትል 3. ለአለርጅ በሽታ ህክምና ክትትል 4. ለላ………….. |  |
| 022 | ለጥያቄ 016 መልሱ አይደለም ከሆነ; ለወደፊት ልትነግርዉ/ልትነግረዉ እቅድ አለሽ/አለክ? | | 1. አዎ  2. አይደለም |  |
| 023 | ልጁ/ልጅቱዋ ስለኤችይቭ ኤድስ ሁነታ/ በስንት ዓመቱ/ዋ ማወቅ አለበት/አለባት ብለሽ/ብለህ ታስባለሽ/ለህ? | | ……………………..ዓመት |  |
| 024 | ለልጆዎች ስለኤችይቭ ኤድስ በሽታዉ መናገር ሀላፊነት ያለዉ/ላት ማን ነዉ? | | 1. እናት 2. አባት 3. አያት 4. የጤና ባለሙያ 5. ለላ……… |  |
| 025 | ልጁ/ልጅቱዋ በኤችይቭ ኤድስ ምክንያት መጥፎ ስም የሚሰጥ ይመስልሻል/ይመስላል? | | 1. አዎ 2. አይደለም |  |
| **ክፍል ሐ፡**  ልጆችና የልጆች አስተማሚ ኤችይቭ ኤድስ ህክምና የተመለከተ ጥያቄ | | | | |
| 026 | የአስተማሚዉ ኤችይቭ ኤድስ ሁነታ ምንድ ነዉ? | | 1. ፖዜቲቭ (ያለበት/ያለባት) 2. ነጌቲቭ (የለለበት) 3. ያልተመረመረ/ች |  |
| 027 | ለጥያቄ 026 መልሱ; ፖዜቲቭ (ያለበት/ያለባት) ከሆነ መድሀኒቱን ጀምርዋል/ጀምረሻል? | | 1. አዎ 2. አይደለም |  |
| 028 | ኤችይቭ ኤድስ ወደ ልጁ/ልጅቱዋ የተላለፈበት ከማን ነዉ? | | 1. ከቤተሰብ 2. ከቤተሰብ ዉጭ |  |
| 029 | ልጁ/ልጅቱዋ በስንት ዓመቱ/ዋ ተገኘበት/ባት? | | ………………..ዓመት |  |
| 030 | በአለም ጤና ድርጅት መሰረት የልጁ/ልጅቱዋ በሽታዉ በየትኛዉ ደረጃ ላይ ይገኛል? | | 1. ደረጃ 1 2. ደረጃ 2 3. ደረጃ 3 4. ደረጃ 4 |  |
| 031 | ልጁ/ልጅቱዋ መድሀኒት ከጀመረ/ከጀመረች ስንት ዓመቱ/ዋ ሁነዋል/ሆናት? | | ………………..ዓመት |  |
| 032 | ስለኤአርት ወይም መድሀኒት ሀላፊነት ያለዉ ማን ነዉ? | | 1. ራሴ 2. ልጁ/ልጅቱዋ |  |
| 033 | ኤአርት ወይም መድሀኒትበትክክል ይወስዳል/ትወስዳለች? | | 1. በጥሩ ሁነታ 2. ምንም አይልም 3. ብዙ ግዜ አይወስድም/አትወስድም |  |
| 034 | ልጁ/ልጅቱዋ ከዚህ በፊት ሆስፕታል ገብተዉ ታክመዉ ያዉቃለል/ታዉቃለች? | | 1. አዎ 2. አይደለም |  |
| 035 | ስለኤችይቭ ኤድስ ለልጅሽ ለመናገር ከጤና ባለሙያ ጋር ተወያይተሸ ታዉቃለሽ/ታዉቃለህ? | | 1. አዎ 2. አይደለም |  |
| 036 | ለጥያቄ 035 መልሱ አዎ ከሆነ፤ በቂ ግንዛቤ አገንተሻል/አገንተዋል? | | 1. አዎ 2. አይደለም |  |
| 037 | ልጁ/ልጅቱዋ ከዚህ ጤና ተቋማት ዉጭ እርዳታ አገንተዋለች/አገንተዋል? | | 1. አዎ 2. አይደለም |  |
| 038 | ለጥያቄ 035 መልሱ አዎ ከሆነ፤ ምን አይነት እርዳታ | | 1 ገንዘብ  2 ምግብ  3 የምክር አገልግሎት  4 ለላ ካለ ይጠቀስ |  |
