# Supplementary material for: HIV-positive status disclosure and associated factors among children in public health facilities in Dire Dawa, Eastern Ethiopia: A cross-sectional study
Source: PLoS One. 2020 Oct 12;15(10):e0239767. doi: 10.1371/journal.pone.0239767 (PMC7549787; doi:10.1371/journal.pone.0239767)
Supplement: S2 Questionnaire — (DOCX) [file pone.0239767.s002.docx]

**Information Sheet**

**DIRE DAWA UNIVERSITY,**

**College of Medicine and Health Science**

**Department of Midwifery**

**RESEARCH AND TECHNOLOGY INTERCHANGE DIRECTORATE**

**HIV positive status disclosure and associated factors among children** **in public health facilities in Dire Dawa, Eastern Ethiopia: A cross-sectional study**

Questionnaire Identification Number ____________

Data collector name is _________________________I am working as in the research conducted by Alemu Guta and his colleagues, who are conducting their research on HIV-positive status disclosure and associated factors among children in public health facilities in Dire Dawa, Eastern Ethiopia, 2019

We would like your honest opinion pertaining to the questions.

**Name of the organization**: Dire Dawa University

**Name of the Sponsor**: Dire Dawa University

**Introduction**

Information sheet and consent form is prepared for caregivers who will be participated in this research project.

This information sheet and consent form is prepared to explain the study you are being asked to join. Please listen carefully and ask any questions about the study before you agree to join.

You may also ask questions at any time after joining the study.

**Purpose**

I am hopeful that this research will assess of HIV-positive status disclosure and associated factors among children to benefit the child health care improvement and quality of care. We will provide each of the units with research results and conclusions for your information.

**Procedure**

You are invited to take part in this project. If you are willing to participate in this project, you need to understand and say ‘yes’ on the agreement form. Then after, you will be interviewed by the data collector. All your responses and the results obtained will be kept confidential by using coding system whereby no one will have access to your response.

**Risk/ Discomfort**

By participating in this research project, you may feel that it has some discomfort especially on wasting time about 30-40 minutes. We hope you will participate in the study for the sake of the benefit of the research result. There is no risk in participating in this research project.

**Benefits**

If you participate in this research project, there may not be direct benefit to you but your participation is likely to help us in assessing of HIV-positive status disclosure and associated factors. Ultimately, this will help us to identify the gap and take the appropriate intervention by the authorized stakeholder. You will not be provided any incentive or payment to take part in this project.

**Confidentiality**

The information collect from this research project will be kept confidential and information about you that will be collected by this study will be stored in a file, without your name, but a code number assigned to it. In addition, it will not be revealed to anyone except the principal investigators and will be kept locked with key.

**Right to refuse or withdraw**

You have full right to refuse from participating in this research. You can choose not to respond to some or all questions if you do not want to give your response. You have also the full right to withdraw from this study at any time you wish, without losing any of your right. Persons to contact: If you have any question to ask, please contact

| Name | | Phone | | E-mail | |
| --- | --- | --- | --- | --- | --- |
| Alemu Guta | | +251945996459 | | [chelsea0061@gmaill.com](mailto:chelsea0061@gmaill.com) | |

**Consent Form**

I undersigning this document, I am giving my consent to participate in the study entitled as “HIV-positive status disclosure and associated factors among children in public health facilities in Dire Dawa, Eastern Ethiopia, 2019” I have been informed that the purpose of this study is to assess prevalence and associated factors of care givers disclosure of HIV diagnosed to their HIV infected children in Dire Dawa. I have understood that participation in this study is entirely voluntarily. I have been told that my answers to the questions will not be given to anyone else and no reports of this study ever identify me in any way. I have also been informed that my participation or non-participation or my refusal to answer questions will have no effect on me. I understood that participation in this study does not involve risks. I understood that Alemu Guta is the contact person if I have questions about the study or about my rights as a study participant. Do you have any question?

Do I have your agreement to proceed? If yes continue, if no .Stop, Thank you!

- Date: ______/_____/_____
- Supervisor’s Name_______________________
- Signature_________

**HIV-positive status disclosure and associated factors among children in public health facilities in Dire Dawa, Eastern Ethiopia: A cross-sectional survey**

**Section A: Socio-demographic characteristics of children and caregivers**

**Instructions:** Write your response on the space provided for open ended questions and Encircle your Response for close ended questions.

| S. no | Question | | Coding categories | Skip | |
| --- | --- | --- | --- | --- | --- |
| 001 | Residence? | | 1. Rural……………. 2. Urban…………. |  | |
| 002 | Age? | | …………………… year |  | |
| 003 | Sex? | | 1.Male  2.Female |  | |
| 004 | Religion? | | 1. Orthodox 2. Muslim 3. Protestant 4. Catholic 5. Other (specify |  | |
| 005 | Ethnicity? | | 1. Amhara 2. Oromo 3. Somale 4. Tigrae 5. Gurage 6. Other |  | |
| 006 | Education status? | | 1. Unable to read and write 2. Able to read and write 3. Elementary 1-8^th^ grade 4. High school 9-12^th^ grade 5. Diploma and above |  | |
| 007 | Occupation? | | 1. Governmental employed 2. Privately employed 3. Student 4. Merchant 5. Housewife 6. Other (specify) |  | |
| 008 | Marital status? | | 1. Single 2. Married 3. Divorced 4. Widowed |  | |
| 009 | What is your relationship with child? | | 1. Biological parents 2. Grand parent 3. Siblings 4. Relative 5. Others |  | |
| 010 | Age of Child? (in year) | ………………. Year | | |  |
| 011 | Sex of child? | 1. Male 2. Female | | |  |
| 012 | Child school grade? | 1. Not started education 2. Primary school (1-8) 3. Secondary school (9-12) | | |  |
| 013 | With whom the child does, currently, live with? | 1. Biological parents 2. Grandparents 3. Siblings 4. Relatives 5. Others | | |  |
| 014 | Has the child lost any of his/her nucleus family members with HIV? | 1. Yes 2. No | | |  |
| 015 | If answer is **yes** for Q=**014;** lost whom? (multiple answer is possible) | 1. Mother only 2. Father only 3. Both mother and father 4. Siblings | | |  |

**Section B**: **HIV positive status disclosure among Children**

**Instructions:** Write your response on the space provided for open ended questions and Encircle your Response for close ended questions.

| Sr.n | Question | Coding categories | Skip |
| --- | --- | --- | --- |
| 016 | Child knows his/her HIV positive status? | 1. Yes…………… 2. No…………….. |  |
| 017 | If answer **yes** for **Q-016**, at what age did you disclose? | At---------------------years |  |
| 018 | Who disclosed about his/her HIV status to the child? (Multiple answers possible) | 1. Mother 2. Father 3. Grand parents 4. Siblings 5. Relatives 6. Health care workers 7. Hear from friends/neighbors 8. Others(specify) |  |
| 019 | Why did you decide to disclose for your child about his/her HIV status? (Multiple answers possible) | 1. Because child thought to be matured 2. Because repeated question of the child what happen to him/her 3. To take or adhere to medications 4. Right to know about his/her disease condition 5. To take care of his/her selves and prevent unknowingly disease transmission 6. To share responsibility and to get relief 7. Other………………… |  |
| 020 | If you didn’t disclose, why you didn’t decide to disclose the child about his/her HIV status? (Multiple answers possible) | 1. Because the child is too young 2. Fear of self-discrimination 3. The child inability to keep secret 4. Future relation with the family would be affected 5. He/she may feel hopelessness 6. Because I am the one that transmit the virus so, I fell guiltiness 7. Fear of emotional and health consequence 8. Lack of knowledge 9. Others (specify)…………….. |  |
| 021 | If you didn’t disclose, what you told the child the reason for visiting health facility? | 1. For TB follow up 2. For heart disease follow up 3. For allergic follow up 4. Others (specify)…………………. |  |
| 022 | Do you have a plan to disclose in the future about his/her HIV status to your child? | 1. Yes 2. No |  |
| 023 | The preferred age at which the child should know about his/her HIV status? | ……………year |  |
| 024 | Who should have the responsibility of disclosing HIV status to the child? (Multiple answer possible) | 1. Mother 2. Father 3. Grand-parents 4. Health workers 5. Others (specify) |  |
| 025 | Do you think the child gets stigmatized due to his/her HIV positive status? | 1. Yes 2. No |  |

**Section C: Clinical characteristics of caregivers and children**

**Instructions:** Write your response on the space provided for open ended questions and Encircle your Response for close ended questions.

| Sr.No | Question | Coding categories’ | Skip |
| --- | --- | --- | --- |
| 026 | HIV status of caregiver? | 1. Positive 2. Negative 3. Not tested |  |
| 027 | Did the caregiver start ART? | 1. Yes 2. No 3. Others(specify) |  |
| 028 | Mode of transmission of HIV to child? | 1. Perinatal 2. Non-perinatal |  |
| 029 | Age at diagnosis of HIV positive status of the child? | ……………….Year |  |
| 030 | WHO clinical stage of the child? | 1. Stage 1 2. Stage 2 3. Stage 3 4. Stage 4 |  |
| 031 | Child duration on ART? | …………..year |  |
| 032 | Who is the responsible for ART? | 1. Caregiver 2. Child themselves |  |
| 033 | How is treatment adherence of the child? | 1. Good 2. Fair 3. Poor |  |
| 034 | Did the child get hospitalized previously? | 1. Yes 2. No |  |
| 035 | Did you discuses about disclosure issue with your child's health care provider? | 1. Yes 2. No |  |
| 036 | If the answer for Question-035 was yes, did the health care provider adequately cover the issues like disclosure? | 1. Yes 2. No |  |
| 037 | Did the child got support from other organizations? | 1. Yes 2. No |  |
| 038 | What kind of support did he/she get? (Multiple answers possible) | 1. money 2. food 3. counseling 4. Others ( specify) |  |
