# Supplementary material for: HIV-positive status disclosure and associated factors among children in public health facilities in Dire Dawa, Eastern Ethiopia: A cross-sectional study
Source: PLoS One. 2020 Oct 12;15(10):e0239767. doi: 10.1371/journal.pone.0239767 (PMC7549787; doi:10.1371/journal.pone.0239767)
Supplement: S1 Certificate — (PDF) [file pone.0239767.s004.pdf]

97

PUBSURE CERTIFIED QUALITY

- Ready to submit (71-100)
- Requires minor revisions (31-70)
- Requires major revisions (1-30)

Ready to Submit

HIV-positive status disclosure and associated factors among children in public health facilities in Dire Dawa, Eastern Ethiopia: A cross-sectional study

Alemu Guta, Habtamu Abera, Kirubel Anteab, Legesse Abera, Abdurezak Umer

Generated: **24/08/2020 19:44 IST**
Source: Manuscript.docx

Statistics

25972 Characters
4396 Words
261 Sentences
29 References

Concepts

AIDS, Antiretroviral drug, Health care, HIV, Health care provider, Ethiopia, Viral load, Tuberculosis, Reverse transcriptase, CCR5

Language Quality

35 errors were detected in 4396 words of the manuscript

Score 57/60

References

29 references in the article
0 self-citations of an author

Score 40/40

TOTAL SCORE

97

Additional report metrics

Tables and Figures

All tables and figures included have been cited in your manuscript.

Inclusive language

The manuscript was checked for racist, sexist, and abusive language. PASS

Ethical declarations

Relevant ethical declarations have been identified. Check your journal instructions if you need these in your cover letter as well.

Commercial declarations

Declarations of commercial interest were identified in your manuscript. Check your journal instructions if you need them in your cover letter as well.

Disclaimer: R Pubsure certificate/report is an assessment of your manuscript against standard submission requirements. The certificate/report is intended only as a manuscript improvement tool and does not guarantee publication in any journal.

Privacy & Site Usage Policy | support.pubsure@researcher.life | Website : www.pubsure.researcher.life
© 2020 Cactus Communications. All Rights Reserved.
